# Barriers and facilitators for treatment-seeking among women with genital fistula: a facility-based qualitative study in Bangladesh

**DOI:** 10.1186/s41182-025-00704-w

**Published:** 2025-02-28

**Authors:** Kanako Kon, Atsuko Imoto, Sabina Faiz Rashid, Ken Masuda

**Affiliations:** 1 School of Tropical Medicine and Global Health, Nagasaki University, 1-12-4 Sakamoto, Nagasaki, 852–8523 Japan; 2 BRAC James P Grant School of Public Health, BRAC University, Floor 10-13, BRAC Tower, 65 Mohakhali, Bir Uttam A K Khandakar Road, Dhaka, 1212 Bangladesh

**Keywords:** Genital fistula, Obstetric fistula, Barriers, Facilitators, Treatment-seeking behavior, PASS model, Bangladesh, Qualitative study

## Abstract

**Background:**

Women living with genital fistula often endure prolonged suffering and face multiple barriers to accessing treatment. Bangladesh’s government has enhanced referral mechanisms, enabling case detection in communities and facilitating surgical interventions at medical college hospitals through nationwide initiatives. However, research on barriers and facilitators for fistula treatment in Bangladesh remains limited. Detailed insights into treatment-seeking paths with time sequences are scarce. This study aimed to explore facilitators and barriers to completing fistula treatment with the description of treatment-seeking paths. This study is important to assist with future policy and program strategies for fistula treatment.

**Methods:**

A facility-based qualitative study was conducted at Dhaka Medical College Hospital, Dhaka, Bangladesh. Data were collected from February to May 2024 through 18 in-depth interviews (IDIs) with in-patients, five IDIs with families, and 11 key informant interviews with health service providers. Participants’ treatment-seeking paths were described chronologically and identified patterns of treatment-seeking paths. Thematic analysis, guided by the Partners for Applied Social Sciences model for health-seeking behavior and access to care, was used to analyze case histories.

**Results:**

The average duration of treatment-seeking by the women was 39 months, with a maximum of 22 years. Women with fistula often sought care at multiple facilities (up to eight), suspended treatment, and encountered systemic obstacles that delayed treatment. Key barriers included scarce information on illness and treatment in the community, less decision-making power, failure of medical communication, and systemic failures in cost, treatment, and referral systems. Facilitators that motivated women to complete treatment included informal peer support through shared treatment experiences and emotional, physical, and financial support.

**Conclusions:**

Analysis of treatment-seeking paths revealed the absence of standardized treatment routes for women with fistula. To ensure effective care, raising societal awareness about fistula, improving treatment and referral systems, enhancing medical communication, and providing peer and emotional support are strongly recommended.

**Supplementary Information:**

The online version contains supplementary material available at 10.1186/s41182-025-00704-w.

## Background

Genital fistula is an abnormal opening between the vagina and urinary bladder or rectum caused by prolonged obstructed labor or pelvic surgery, leading to uncontrollable urine leakage and fecal incontinence [1, 2]. Although reliable prevalence data are limited [3], the World Health Organization (WHO) estimates 500,00 to 100,000 new cases annually, with more than 2 million women living with fistula particularly in Africa and Asia [4, 5]. More than 90% of pregnancies associated with fistula result in stillbirths [6, 7]. Affected women experience social exclusion and abandonment due to the foul odor of excrement leakage [6, 8, 9]. Additionally, incontinence restricts access to employment, preventing full work capacity [7]. Fistula increases neonatal mortality, reduces quality of life, and causes significant socio-economic consequences, despite being treatable by surgery [9, 10].

In 2003, the United Nations Population Fund (UNFPA) launched the “Campaign to End Fistula”, for elimination of fistula by 2030 [11]. The “South Asia Conference for the Prevention and Treatment of Obstetric Fistula” was held in Bangladesh the same year to introduce the UNFPA’s fistula campaign to South Asia [12]. Bangladesh demonstrated strong commitment by completing a nationwide needs assessment of the fistula situation and planning to establish South Asia's first National Fistula Centre [12]. The estimated adjusted prevalence rate in Bangladesh is 38 per 100,000 women aged 15–49 years (90% uncertainty interval [UI] 25–58), with approximately 13,376 (90% UI 8,686–20,112) women of reproductive age affected [13].

Women with fistula endure lengthy periods before receiving hospital treatment, facing numerous barriers. A 2003 analysis revealed that 12 of 27 women had lived with fistula for more than 10 years due to limited knowledge of treatment availability, financial constraints, and lack of caregivers to accompany them [12]. Biswas et al. also reported that the mean duration of fistula in Moulvibazar district, Sylhet division, was 19 years [14]. In 2012, Blum et al. revealed treatment-seeking experiences among four potential fistula cases detected through screening by household visits in rural areas in Bangladesh [15]. These individuals pursued care repeatedly but ultimately resorted to home remedies after encountering unsuccessful surgeries, high costs, and misinformation about treatment-eligible facilities [15]. Similarly, Biswas et al. noted that only one of five women accessed tertiary-level care but could not continue due to financial barriers [14]. These studies underscore that incomplete treatment is often caused by unresolved symptoms, financial burdens, and misinformation.

**Fig. 1:**
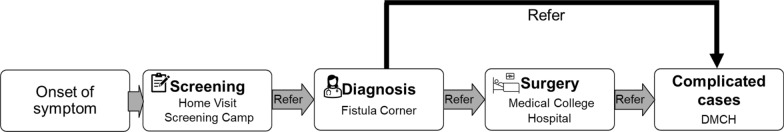
Fundamental referral process for women with fistula

Nationwide efforts, including subsidized treatment, fistula prevention, and patient identification initiatives, aim to achieve a fistula-free status [16, 17]. The Ministry of Health and Family Welfare (MOHFW) has developed strategies to strengthen the health system for fistula prevention, patient detection, and treatment through coordinated referral systems (Fig. 1) through several nationwide projects [18]. Community health workers and fistula screening camps, which is organized by government occasionally, screen suspected cases and refer them to hospitals [18﻿]. Diagnosis is conducted at designated “Fistula Corners”, which have been established in selected district hospitals across Bangladesh. Women diagnosed with fistula are referred to the nearest medical college hospital or other eligible facilities equipped with fistula treatment departments [20﻿]. For cases involving complex fistulas, patients are further referred to the National Fistula Centre (NFC) at Dhaka Medical College Hospital (DMCH). Some private hospitals also play a role in the diagnosis and surgical management of fistula.

However, existing research on detailed treatment-seeking paths among women with fistula is primarily limited to African countries. The three studies in Bangladesh discussed above recruited few participants and lacked time sequenced analyses of treatment paths. Moreover, no studies have explored barriers and facilitators in treatment-seeking after the implementation of nationwide fistula projects in Bangladesh. This research aims to identify patterns in treatment-seeking paths among women with genital fistula and explore facilitators and barriers to complete fistula treatment. In this study, ‘treatment completion’ for genital fistula was defined as the point at which surgeons complete all possible treatments. This study can contribute to finding effective approaches and strategies for promoting fistula treatment.

## Methods

### Study design, setting and period

This study employed a facility-based qualitative approach, appropriate for gaining an in-depth understanding of treatment-seeking experiences among women with genital fistula. Eighteen in-depth interviews (IDIs) were conducted with women with fistula and five family members to capture their experiences. Eleven supplementary informants familiar with fistula treatments and health systems were included as key informants to contextualize the IDI findings.

Data were collected from February to May 2024. The study site was the NFC at DMCH, Bangladesh's oldest public tertiary-level hospital, which serves as a nationwide referral hub for government healthcare services. Established in 2009 with UNFPA Bangladesh's support, the NFC specializes in fistula treatment [19]. While other medical college hospitals in Bangladesh perform fistula repair surgeries, complicated cases are referred to NFC [15].

### Study participants and recruit methods

Twenty-three participants for IDIs and 11 key informants were recruited (Table 1).

**Table 1:** Number of study participants

IDIs	**23**
Women diagnosed with fistula	18
Family	5
KIIs	**11**
Health service providers at DMCH	
Doctor	2
Nurse	3
Administrator	1
Officer at Social Welfare Department	1
Others	
Homeopathic doctor	2
Former president of Obstetrical and Gynecological Society of Bangladesh	1
Technical officer at UNFPA Bangladesh	1

Bold represented the total number of In-depth Interviews (IDIs) and Key Informant Interviews (KIIs)

#### IDIs

Women with genital fistula admitted to the NFC were recruited for IDIs. Participants were purposively selected with following inclusion criteria are: (1) diagnosed with genital fistula caused by obstetric and iatrogenic, (2) aged ≥ 18 years, (3) preoperative or postoperative patients, and (4) consent to participate. Exclusion criteria were (1) difficulty speaking or communicating and (2) scheduling conflicts due to physical examinations or surgeries on the interview date.

The cause of fistula was categorized into two types: obstetric and iatrogenic. Obstetric fistula was defined as abnormal hole caused by prolonged and obstructed labor during delivery before any symptoms were recognized [2]. If a woman had a history of prolonged and obstructed labor during delivery before recognizing any fistula symptoms, the fistula was classified as obstetric. Iatrogenic fistula was defined as the abnormal hole resulting from pelvic surgeries, particularly gynecological procedures, before symptoms appeared [20]. In cases where participants experienced both prolonged obstructed labor and cesarean sections, the fistula was classified as obstetric. Fistula causes were cross-checked using hospital records at NFC to ensure accuracy.

Additionally, the IDIs included five family members of women with genital fistula, including two mothers, one brother, one son, and one daughter-in-law, were recruited. Family members were defined as those accompanying or staying with the participants. Families agreeing to participate and aged ≥ 18 years were included. Participants for IDIs were purposively selected from women in the NFC’s preoperative and postoperative units. When introduced by the healthcare staff at the NFC, the local research assistant explained the study objectives and obtained consent face-to-face in Bengali. Family members were invited to participate if present at NFC on the interview date.

#### KIIs

Key informants involved in fistula treatment, as well as technical partners and an officer of the funding international organization, participated in the study (Table 1). Eleven key informants were purposively recruited, including health service providers at DMCH (one NFC doctor, one outpatient obstetric gynecology doctor, three NFC nurses, one administrator, and one officer from the social welfare department), two homeopathic doctors working in Dhaka's public and private sectors, one former president of the Obstetrical and Gynecological Society of Bangladesh (OGSB), and one UNFPA Bangladesh technical officer. NFC doctors and nurses were approached directly by the local research assistant. Other DMCH service providers, including those from the obstetric and gynecological outpatient and social welfare departments, were introduced by DMCH's assistant director. Informants unaffiliated with DMCH were contacted via email or phone by the principal investigator (PI: K.K) or research assistant, and informed consent was obtained through in-person visits. Homeopathic doctors near DMCH were approached, and those who consented were included. English-speaking informants were interviewed directly by the PI without the local research assistant.

### Data collection procedures

**Fig. 2:**
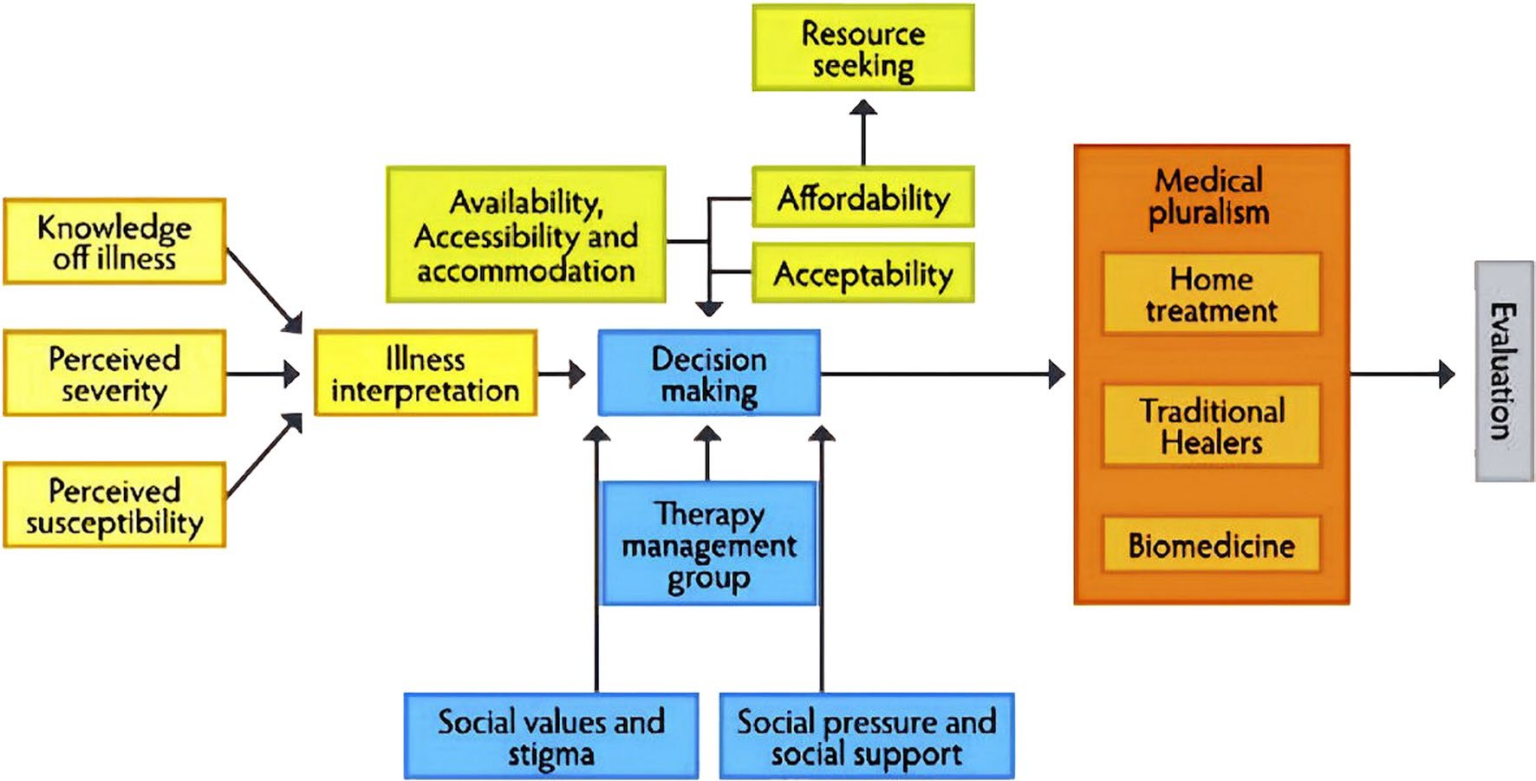
The PASS-model for health-seeking behavior. Source: Hausmann Muela et al. [21]. Permission to use this figure was kindly granted by the author

The PI developed interview guidelines for IDIs and KIIs in English, which were then translated into Bengali and back-translated into English for quality assurance. IDIs employed open-ended questions based on the elements of the Partners for Applied Social Sciences (PASS) model (Fig. 2), theoretical framework [21]. This framework is suitable for describing the processes that individuals undertake to seek care, as well as the elements that hinder or facilitate access to and receipt of treatment [21]. This model incorporates components from other health-seeking behavior models into a cohesive and all-encompassing framework [21]. The KIIs were semi-structured to gather information on case identification, referral systems, challenges in treatment access, and experiences.

Prior to starting data collection, the local female assistant received guidance from the PI regarding fundamental knowledge of genital fistula (cause and type of fistula, diagnosis, and treatment), research objectives, and how to conduct IDIs with practice. The research assistant majored in anthropology and had experience conducting qualitative interviews related to sexual and reproductive health. Pilot tests were conducted with one woman with fistula and one key informant (nurse) at NFC to refine the interview guide and ensure feasibility in practice. The data saturation is defined as the point at which no more new pattern of fistula treatment-seeking path [22]. We aimed to conduct interview with around 20 women with fistula, reflecting previous research on saturation [23]. Observations were carried out by the local research assistant, and the findings were shared during online meetings.

KIIs are conducted face-to-face in the participants' preferred language, either Bengali or English; one KII was conducted online due to scheduling difficulties. IDIs were conducted in Bengali individually. PI checked data quality through transcription and information from the assistant’s observations. If any unclear data were found by the PI, the local research assistant conducted additional interviews, depending on the participants’ availability.

Each interview lasted 30–90 min, depending on participant availability. Participants were allowed to choose their preferred interview location, ensuring privacy, whether in a private space or at their bedside. If adverse psychological reactions would result in stopping the interview and informing DMCH healthcare providers. However, no adverse reactions were observed during data collection. Interviews were audio-recorded with participant consent.

### Data analysis

The collected data were transcribed in Bengali and translated into English. Data analysis was conducted during data collection in a flexible and iterative manner. The analysis comprised two phases.

The first phase involved creating case histories to describe the treatment-seeking paths of women with fistula and identify patterns. Each participant’s treatment-seeking path was described chronologically, beginning with the recognition of the symptom and continuing to their current situation. This included the facilities or individuals they sought care from, the behaviors of participants or their families, and the care received. These paths were categorized into patterns based on shared traits and challenges.

In the second phase, thematic analysis by Guest et al. [24] was applied to identify facilitators and barriers to completing fistula treatment. The PI reviewed the English transcripts multiple times to familiarize the data. The PI conducted the coding process, using qualitative analysis software, MAXQDA Plus 2022. Deductive coding was conducted based on a pre-designed codebook that reflected the theme from the PASS model (Fig. 2), and inductive coding from the collected data was conducted. Key information was categorized into themes from the generated codes.

Information from KIIs was described with data from IDIs as supplementary information. The data collected through women’s and families’ IDIs were compared with information from KIIs. If any information gap was found, the gaps were described by exploring the reasons for the information gaps between service users and service providers.

## Results

### Characteristics of participants for IDIs

Table 2 presents the sociodemographic characteristics of women with fistula who participated in IDIs. Most participants were in their 30s to 40s (8 participants, 44.4%), followed by those under 30 (5 participants, 27.8%). The oldest participant was 50 years old. Participants were from five divisions (Barisal, Chittagong, Dhaka, Khulna, Sylhet), excluding Rajshahi, Rangpur, and Mymensingh. Six women (33.3%) had no formal education or dropped out of primary school, while eight (44.4%) completed primary school. Two women had education beyond a bachelor’s degree. Fifteen (83.3%) were housewives. Eleven participants had fistula caused by iatrogenic reason, whereas six was due to obstetric reason.

**Table 2:** Sociodemographic characteristics of women with fistula

	Number	Percentage (%)	Minimum	Maximum
Age			23	50
< 30	5	27.8		
30–40	8	44.4		
> 40	5	27.8		
Address (division)				
Barisal	2	11.1		
Chittagong	6	33.3		
Dhaka	7	38.9		
Khulna	2	11.1		
Sylhet	1	5.6		
Educational level				
No formal education	4	22.2		
Less primary	2	11.1		
Primary complete	8	44.4		
Secondary complete	2	11.1		
Higher than secondary	2	11.1		
Occupation				
Housewife	15	83.3		
Employee	2	11.1		
Others	1	5.6		
Age of marriage			13	24
< 18	10	55.6		
18–19	3	16.7		
> 20	5	27.8		
Age of first delivery			14	25
< 18	7	38.9		
18–19	3	16.7		
> 20	8	44.4		
Admission status				
Postoperative	7	38.9		
Preoperative	11	61.1		
Cause of fistula				
Obstetric	6	33.3		
Iatrogenic	11	61.1		
Not sure	1	5.6		

### Characteristics of women with fistula in three patterns of treatment-seeking path

**Fig. 3:**
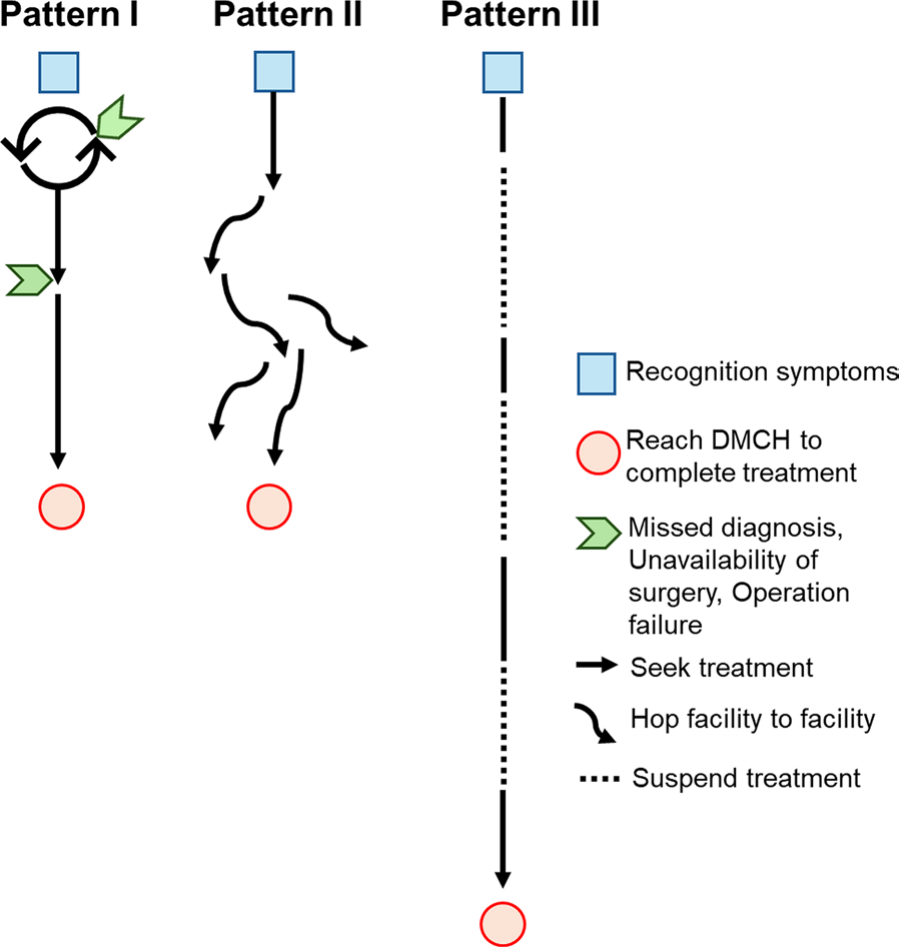
Three patterns of treatment-seeking paths among women with fistula

Three treatment-seeking path patterns (Fig. 3) were identified from the participants' treatment paths. Each participant’s treatment-seeking path is detailed in Additional File 1 [see Additional File 1]. Table 3 summarizes the characteristics of women from IDIs based on these patterns.

**Table 3:** Characteristics of women with IDIs depending on treatment-seeking patterns

	Pattern I^*1^	Pattern II^*2^	Pattern III^*3^
Number of women	11	2	5
Average age	35.4	32.0	38.6
Education			
None or primary uncompleted	4	0	2
Primary completed	5	0	3
Secondary completed	2	0	0
Above	0	2	0
Cause of fistula			
Obstetric	3	0	3
Iatrogenic	7	2	2
Not sure	1	0	0
Duration and number of visited facilities			
Average duration living with fistula with range (months)	5.7 (2.5–10)	5.5 (6–8)	126.2 (10–264)
Average number of visited facilities with range	3.5 (2–6)	7.0 (6–8)	4.4 (3–6)

^*1^: Treatment-seeking with adherence

^*2^: Multiple consultations

^*3^: Prolonged treatment-seeking

#### Pattern I of the treatment-seeking path: treatment-seeking with adherence

Women categorized as Pattern I adhered to medical advice and sought treatment at DMCH within months due to unavailability of local surgical options or failed initial fistula repair surgeries. Eleven cases were identified under this pattern (Table 3), all involving individuals with low education levels and more iatrogenic than obstetric fistula. On average, they lived with the fistula for 5.7 months (range: 2.5–10 months) and visited 3.5 facilities (range: 2–6).

Those categorized as Pattern I sought care within days after recognizing symptoms such as urine leakage or fecal incontinence. Eight participants returned to the hospital where pelvic surgery was performed or where they were initially admitted before noticing symptoms. The other two sought treatment from Kabiraj^1^or homeopathic^2^ practitioners, but returned to facilities within days due to lack of symptom improvement or care refusal.

The background of treatment-seeking is catheterization after surgery. Usually, after pelvic surgery, including cesarean section, participants underwent urethral catheter insertion for several days. Almost all participants recognized their urine leakage after removing their urine catheters. Women perceived that leakage occurred somehow with the current pelvic surgery.

“*It* (catheter) *was installed for 10 days, the doctor took it out when they sent me home. After coming home, I saw my urine leaking. Then again took me to where the Caesar* (cesarean section) *was done.*” (Woman-15, postoperative)

*“It was leaking on its own. I thought it had something to do with the complexity of my cesarean operation.”* (Woman-3, preoperative)

The women adhered to the doctor’s advice following the prescribed medication and catheterization. The maximum duration of medication and catheterization before the first surgery for fistula was six months and five months, respectively. Despite not observing any improvement during this initial treatment attempt, they continued seeking the doctor multiple times and complied with instructions. After the absence of symptom improvement, unavailability of surgery, or failed surgery, the doctor recommended visiting DMCH after several months.

#### Pattern II of the treatment-seeking path: multiple consultations

Two participants were classified as Pattern II with an average age of 32 years (Table 3), younger than those in the other groups. Both participants had bachelor's degrees, indicating a higher level of education. This group visited the most facilities during treatment, averaging seven (range 6–8).

Participants in this pattern sought care at multiple hospitals, frequently moving between facilities. Initially, doctors diagnosed conditions such as urinary infections or attributed symptoms to normal postoperative reactions. However, prescriptions and catheterization did not alleviate urinary incontinence. Seeking improvement, they visited numerous facilities, consulting trusted doctors and factoring in reputations. Key reasons for consulting multiple hospitals included affordability, quicker management, and flexibility in scheduling admission and surgery.

*“I requested him* (doctor) *a lot that he perform the operation on me in a few days, as my job is for life insurance, in a financial institution. Money matters are a lot. Since it is now December, I was saying to perform the operation within that time. But I did not get any response from them* (doctors) *even after a week of admission”* (Woman-8, postoperative)

Women eventually struggled with an enormous burden of payment. The women visited multiple facilities and spent substantial amounts on medications and diagnostic tests due to the high costs of surgery, the unavailability of experienced surgeons, and the lack of available inpatient beds. Several private hospitals quoted fistula surgery costs ranging from 40,000 BDT (340.40 USD) to 80,000 BDT (680.80 USD), amounts they could not afford.

*“Going from one doctor to another in the hospital, I am exhausted from undergoing many tests. I am very scared about how to gain admission and what to do. Besides it is also very painful for me. After I went to* (Doctor A)*, she asked me for a lot of fees, it was very painful for me. Having already spent a lot of money, I wonder how I will I afford the surgery. It seems that I may not be able to have the surgery.”* (Woman-9, postoperative)

Eventually, they arrived at DMCH, exhausted and unable to access care at the other hospitals they visited, and finally underwent surgery.

#### Pattern III of the treatment-seeking: prolonged treatment-seeking

Pattern III included five women with an average age of 38.6 years (Table 3), higher than the other groups. Education levels were lower, with two women being uneducated or having incomplete primary education and three completing primary education. The average duration of living with fistula was 126.2 months (10.5 years), ranging from 10 to 264 months (22 years).

All women in this group sought treatment throughout their journey but suspended it at various stages. They did not follow doctors’ recommendations for subsequent visits due to experiences such as a lack of symptom improvement after prescribed medication, initial catheterization, fistula surgery, or being told to wait months for surgery.

Furthermore, women’s treatment efforts were hindered by their husbands' opposition or neglect.

*“My husband did not take me* (for the treatment)*. Besides I needed money, and I do not know anything myself”* (Woman-10, preoperative)

A woman’s son described his mother’s experiences as follows:

*“For 20 years, the problem remained the same because my father, as the head of the family, decided everything. When my father refused to do the operation, no further treatment took place.”* (Family-4, Woman-5’s son)

These women lacked decision-making power regarding treatment, with husbands primarily deciding their actions.

Nevertheless, the women did not abandon their desire for treatment. When they did not pursue biomedical care, they were not inactive but turned to widely available and affordable alternative remedies in Bangladesh, such as homeopathic medicines and religious treatments by *Kabiraj*. According to homeopathic doctors in KIIs, more women preferred homeopathy because of its lower cost compared to biomedical treatments. Women unable to access medical care often found solace in their faith, trusting in Allah’s blessings. One woman paused her treatment for nearly 20 years due to her husband’s opposition, instead seeking traditional remedies locally without informing her family.

Interviewer: “*From whom did you consult the water and Pani-pora,*^3^* Tel-pora*^4^* from the Hujur*^5^*?*”

Interviewee: “*No one suggested that particularly, I took them on my own, believing in Allah.*” (Woman-5, preoperative)

Eventually, the participants resumed treatment and reached DMCH. Three key factors motivated them to seek treatment again: first, when families or relatives provided financial support and facilitated access to appropriate hospitals; second, when severe symptoms such as pain or abdominal edema emerged; and third, when they learned about successful treatments for similar cases at DMCH.

Interviewee: “*I asked people if there was a cure for this disease. No one in my area knew anything. Now I have come here* (DMCH) *after hearing from one of my aunties who had such a problem.*”

Interviewer: “*Did she come to Dhaka Medical College and get better?*”

Interviewee: “*Yeah. She was cured 10 years ago.*” (Woman-12, postoperative)

Hearing about successful treatment outcomes in similar cases was a significant motivator for women to resume their treatment journey.

### Barriers and facilitators to seek treatment

**Fig. 4:**
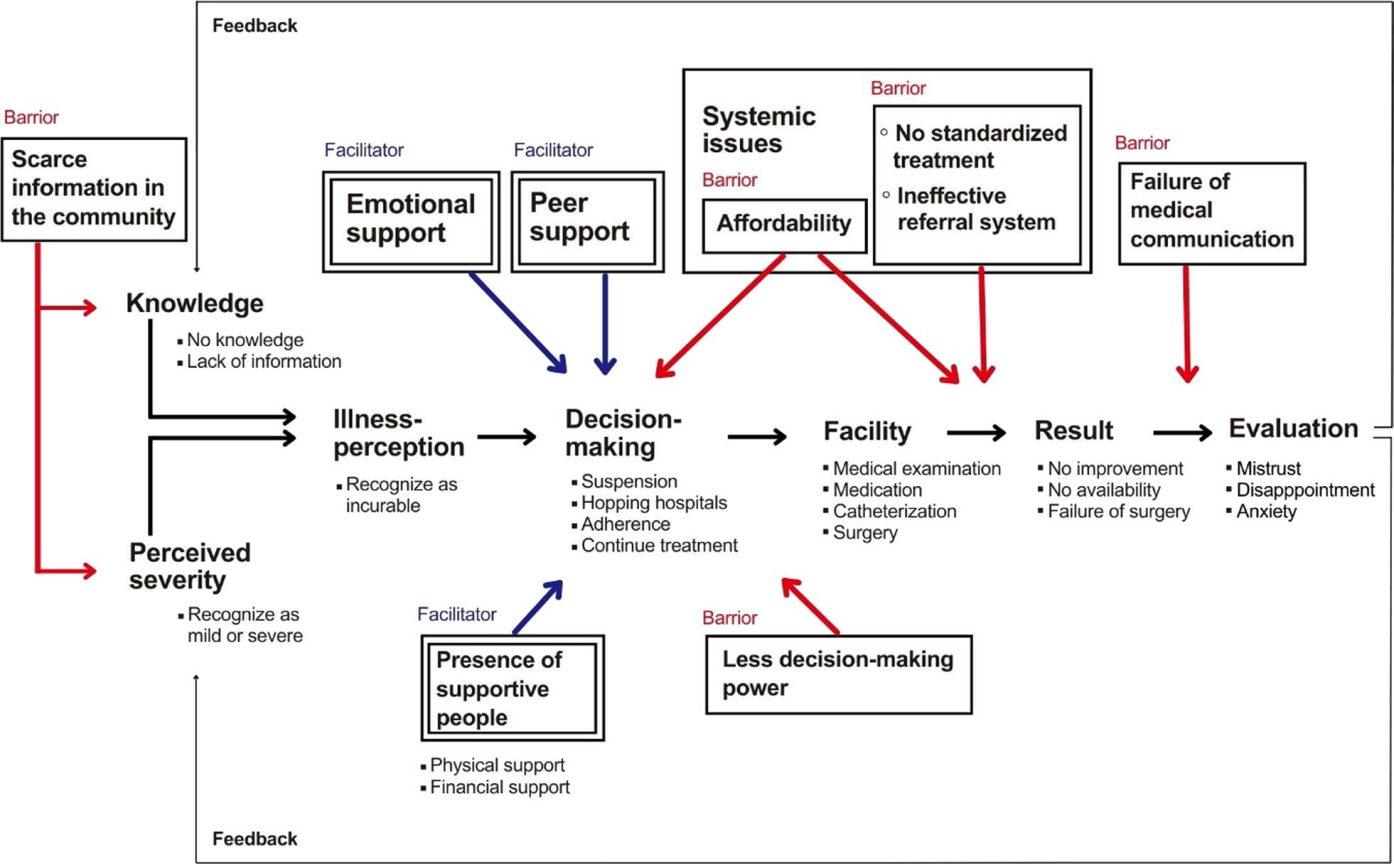
Cycle of the treatment process with barriers and facilitators to complete treatment

Figure 4 shows the cycle of the treatment process, highliting the barriers and facilitators identified through thematic analysis. Women with fistula followed a recurring cycle, transitioning from illness interpretation to treatment evaluation, often repeating this process.

Due to limited information and knowledge about fistula, some of the women believed the condition was incurable. Women’s perceptions of symptoms varied, with some viewing them as mild and others as severe, as none had encountered similar cases before. Decision-making was further influenced by both barriers and facilitators, including affordability, women’s less decision-making power, emotional and peer support, and the presence of people who provided physical and financial support. Negative outcomes, such as no improvement in symptoms and failed surgeries, influenced their evaluation and subsequent attempts to seek treatment.

#### Scarce information on illness and treatment in the community

The first barrier was a lack of knowledge regarding the fistula and its curability. Information on illness and its curability has not been widely disseminated in the community. Several key informants indicated that information regarding fistula now reaches the community through radio, TV, and social media. However, most women and their families remained unaware of fistula.

“*I’ve never seen anyone have this problem before, so I cannot imagine it, it is just my bad luck.*” (Woman-16, postoperative)

Not only women, but also the community perceived the illness as incurable due to limited information, affecting women’s motivation for treatment.

“*We don’t have people around us who understand this illness. Village people or people in my area do not know about diseases or where to go for treatment.*” (Woman-11, preoperative)

“*The people in the village say that this disease was incurable. That’s why I was breaking down mentally.*” (Woman-7, preoperative)

Limited information about illness and available treatment influenced women's treatment-seeking behavior, leading to delayed treatment.

“*I did not get any information about the treatment, so I did not even consider getting the treatment quickly.*” (Woman-12, postoperative)

Thus, scarce information about illness and treatment availability in the community affected women’s behavior regarding treatment completion.

#### Less decision-making power

The family’s influence on treatment-seeking was significant, often leaving women without decision-making power regarding their treatment.

“*I needed to see a doctor much earlier. Many time I brought money to my husband from my parents for my operation. I don’t know anything, I don’t know. Every time I brought money, he said he would take me. But my husband did not bring me, he spent all his money to get drunk, I do not understand anything outside, otherwise I would have tried to show myself* (I would have tried to see a doctor by myself).” (Woman-10, preoperative)

Few women independently manage their finances. All the women in the treatment Pattern III relied on their families to manage household finances. Male family members controlled financial resources and required women to seek family permission to pursue medical treatment. Although these women expressed a desire for care, they could not visit healthcare facilities without the consent of family members who held the decision-making authority.

#### Affordability of treatment with the collapse of service delivery

Unaffordability of treatment emerged as the primary barrier for women and their families. Despite claims for free fistula surgery at several hospitals, women still faced costs for diagnostic tests, medications, and food.

“*There are many challenges regarding financial conditions. This is that I am coming here* (DMCH) *with loans and doing treatment. Not everyone always has money, and loans are not always available. I have many problems with money.*” (Woman-7, preoperative)

Most participants reported taking loans for medications and diagnostic tests. A discrepancy existed between KIIs and IDIs regarding the costs of tests and medications at DMCH. DMCH healthcare providers stated that diagnostic tests were affordable and medications were nearly free. Additionally, the DMCH social welfare department subsidizes treatment costs for patients struggling to pay, based on a doctor’s referral. However, patients reported paying between 1,650 and 20,000 BDT (14.03–170 USD) for medications and between 2,000 and 12,000 BDT (17–102 USD) for tests.

Several women described high expenditures due to the existence of *Dalal*,^6^ a local term referring to medical brokers. These brokers are employed by private facilities, such as diagnostic center, to persuade or redirect patients from public hospitals to utilize their services, for which they receive a fee from the respective facilities [25]. The existence of *Dalal*s greatly impacted women’s expenses.

“*There are many Dalal in the outdoor area of ​​Dhaka Medical* (DMCH)*. They harass during the examinations, fidgeting with papers. Some say it will take 5000 BDT* (42.5 USD), *some say 3000 BDT* (25.5 USD)*. I have to do this work by dragging. Faced a problem with this.*” (Woman-11, preoperative)

Informant: “*When I first came here, a Dalal took 500 taka* (4.26 USD) *to do the test. A woman prescribed the test to the doctor, X-rays are being done there, I am doing ECG, blood test, urine test. 10,000 taka* (85.10 USD)* were taken for these.”*

Interviewer: *“Where did you get the tests?”*

Informant: *“From the diagnostic center. They took more money from us for all the tests there. But they were not useful in Dhaka Medical* (DMCH)*. All the tests had to be repeated.”* (Woman-14, postoperative)

The diagnostic tests instructed by *Dalal*s were often not useful at DMCH, resulting in wasted money and duplicate payments.

The termination of the fistula project resulted in treatment interruptions and higher costs compared to the phase during which the project was actively running. One woman noted that her initial surgery was nearly free due to UNFPA funding, but after the project ended, her financial burden grew.

“*Since now, I have to pay my own money for the treatment, so it took some time to prepare for this. Before everything was done for free, but now you have to pay for everything yourself. And now any kind of tests and medicines cost much more than before.*” (Woman-2, preoperative)

A KII with a UNFPA Bangladesh officer revealed that funds were redirected from individual support to a government-led health system strengthening for fistula care. This shift prioritized cost-effectiveness and sustainability, but made treatment costs less affordable for women than before.

#### No standardized treatment and ineffective referral system

A third barrier was the lack of standardized treatment for genital fistula and a functioning referral system.

Some doctors failed to promptly diagnose fistula, leading patients and their families to believe that the leakage was a temporary postoperative reaction.

*“When we first see the problem, we consulted the person who performed the operation. She said that, it is no problem, so we thought it was no problem.”* (Family-5, Woman-16’s daughter in law)

“*I come home, eat, sleep, wake up and see that my clothes are wet. I told my daughter to call the doctor. She called later, and the doctor said there would be no problem, you bring her. Then I went* (hospital) *after five days… After going, he* (the doctor) *prescribed medicine, and said nothing would happen, how long will it be like this, you will be fine by taking medicine.”* (Woman-17, preoperative)

These instructions caused the women to recognize their symptoms as mild or to harbor doubts about doctors when no symptom improvement was observed.

According to the former president of the OGSB, Bangladesh lacks national treatment guidelines for genital fistula. Fistula surgeons rely on the international guidelines of the International Federation of Gynecology and Obstetrics or a book authored by a prominent Bangladeshi fistula surgeon. Key informants reported varying durations of conservative treatment, indicating non-surgical approaches for fistula by urinary catheterization before surgery. While one recommended three months, another suggested three to six weeks. Several participants noted being instructed to wear a urinary catheter for three months. However, some struggled to manage urine leakage from the catheter.

“*They gave me another pipe for three months. But sister* (interviewer)*, it is a lot of trouble. The tube area was always burning. I am a poor woman. It was very painful. The urine did not flow into the tube. They gave me medicine for three months. After taking it, I saw nothing was going right. Then I opened the tube again with the big doctor* (another doctor at a private hospital in her local area).” (Woman-18, preoperative)

Despite following medical advice, including catheterization and medication, some women developed mistrust in healthcare providers when symptoms persisted.

“*It didn’t go away even after taking medicine for this. After taking the doctor's medicine, it did not decrease a bit. That's why the doctor gave me another medicine. It has not decreased even a bit. The price of that medicine was 300 taka per ten. My husband is a poor man. It is not reducing even after taking such expensive medicine. I was very upset because of that.*” (Woman-14, postoperative)

Women often underwent extended periods of medication and catheterization before being referred to DMCH when no improvement was observed.

While medical college hospitals had efficient referral systems with formal referral letters, rural hospitals often failed to refer women for surgery promptly. Some surgeries were delayed due to complex fistula and inadequate resources, leading to eventual referrals to DMCH. In addition, some doctors advised waiting three to six months for surgery, causing prolonged delays and multiple hospital visits.

#### Failure of medical communication

Failure of medical communication indicated inadequate counseling by healthcare providers about fistula and its treatment process. The lack of communication between women with fistula and healthcare providers contributed to misunderstandings, unrealistic expectations, and raised anxiety, which hindered treatment-seeking progress. Many women did not fully understand the fistula and treatment process before arriving at the DMCH due to insufficient explanations.

“*The doctor said it was fistula. But they did not say anything elaborately.*” (Woman-8, postoperative)

A woman’s daughter-in-law, a former nurse, shared their experiences.

“*I am actually trying to give her* (the woman with fistula) *a lot of support mentally, saying that it is not a big problem. But she* (the woman with fistula) *was very scared, we were also afraid of what the real problem was! Then, for 2–3 months, the doctors did not tell us what the problem was. They were just prescribing medicine, but we were not getting any solution, and we were also very tense.*” (Family-5, Woman-16’s daughter-in-law)

Some women misunderstood that preoperative medications and catheterization would cure the fistula rather than manage symptoms. Disappointment over a lack of post-treatment improvement led some to discontinue treatment. A nurse at DMCH explained that staff provide careful counseling about the treatment schedule, as some women wish to expedite the process. However, most women arrived at DMCH without prior counseling or knowledge of the fistula and treatment procedure.

### Facilitators

#### Peer support

Learning from individuals with similar conditions encouraged women to seek and continue treatment despite initial surgical failures or lack of symptom improvement.

“*I took treatment here* (DMCH) *once before. Many people come here for treatment. If it does not work the first time, come three or four times. Then maybe get well. That's why I came here again. I stayed in regular contact with a patient *(a fistula survivor) *by phone. I learned from her that she has recovered. We were here together back then. Then I came to know that 4–5 patients have recovered. Not everyone heals the first time. Some see results* (healed) *after three or four treatments. That is why I came here again after waiting so long.*” (Woman-11, preoperative)

Women received information about fistula treatment from others with similar experiences, motivating them to continue treatment even after the failure of the first surgery.

Women felt afraid and anxious, recognizing they had a rare condition they believed no one else experienced. However, at DMCH, they discovered many peers facing the same challenges.

“*I only thought that I might not recover, later when I came to Dhaka Medical, I saw that there are many patients like me. All of them are undergoing treatment, and when I came to the post-operative* (unit)*, I saw that many are recovering. Then my hope increased. If I receive treatment, I will recover. I have been with hope for so long, I am still doing well, I will understand the rest* (whether I will fully recover) *after catheter is removed.*” (Woman-16, postoperative)

Recognizing peers positively influenced women and motivated them to seek a cure. Peer support played a significant role in encouraging women to complete their treatment.

#### Emotional, physical, and financial support

Women could seek treatment through informal emotional support and the help of individuals who manage their treatment physically and financially.

Despite negative experiences, emotional support—particularly from husbands—kept women motivated to pursue treatment.

“*When I was being rejected like this from everywhere, I was very broken mentally, I was disappointed, then my husband supported me. He said, let's go to Dhaka Medical* (DMCH) *and see what the situation is there.*” (Woman-9, postoperative)

Not only the husband, but also the surrounding people’s acceptance of the women’s situation through encouragement, sympathy, and recommendation to see a doctor supported women’s behavior to seek treatment.

Physical support was also a critical facilitator, including assistance with dietary management during hospitalization, escorting women to the hospital, and helping with housework and childcare.

Financial support was crucial in enabling treatment completion. While many women had limited financial independence within their families, relatives covered treatment costs through savings or loans. Some received financial assistance from family members, such as brothers, even when their husbands opposed hospital treatment.

## Discussion

This study identified three primary treatment-seeking patterns and elucidated various barriers and facilitators affecting fistula treatment completion. Women with fistula encountered significant challenges in accessing treatment through standardized pathways, frequently resulting in delays, movement between healthcare facilities, and treatment suspension. The barriers to completing treatment encompassed scarce information on illness and treatment, less decision-making power, systemic failures in cost, treatment, referral systems, and failure of medical communication. Facilitators comprised peer support and emotional, physical, and financial support, indicating the presence of individuals who managed women’s treatment.

Several studies have described treatment experiences for women with fistula in Bangladesh; however, there is limited description of detailed treatment-seeking pathways and patterns from the recognition of fistula symptoms to the completion of treatment. This study reports comprehensive treatment-seeking pathways and highlights the situations in which participants deviated from the standardized pathways for fistula treatment. The barriers extracted in this study align with findings from previous studies, which also noted affordability issues, missed diagnoses, and ineffective referral systems, alongside facilitators such as peer support and psychological, physical, and financial assistance [14, 15, 26, 27]. Despite the implementation of several nationwide initiatives in Bangladesh, barriers to completing fistula treatment remain largely consistent with previous studies and continue to impact women.

The uniqueness of this study lies in finding that external barriers rather than personal own beliefs or perceptions, influenced treatment-seeking behavior. Previous studies in African countries reported that most women concealed symptoms and initially sought traditional treatments [28–31]. Women with fistula in Ghana emphasized that personal beliefs affect treatment-seeking, delaying treatment [32]. However, participants in this study were motivated to seek biomedical treatment promptly after recognizing symptoms. Among the 18 participants, 16 attempted to seek hospital treatment within 1 month of recognizing symptoms. Certain female patients initially sought traditional remedies, but subsequently transitioned to biomedical treatment due to rejection by traditional healers or persistent symptoms. Additionally, some participants sought traditional and religious remedies as alternatives due to family opposition and lack of financial support. This highlighted that women in this study were more influenced by external barriers, such as financial challenges due to family opposition to seek treatment.

### Effective community approaches to raise awareness

Limited information about the illness and treatment hindered women’s efforts to seek care. Several participants reported having never encountered similar cases, leading communities to perceive the condition as incurable and demotivating women from pursuing treatment. A systematic review also identified lack of awareness as a significant barrier to fistula treatment [26]. Previous research in Uganda highlighted the effectiveness of radio announcements in promoting early treatment-seeking [31]. In Bangladesh, the “Fistula Care Plus” project used various media channels, including electronic, print, radio, television, and workshops, to raise awareness [17]. According to key informants, social media is now used to share information about fistula and available treatments. However, critical information often fails to reach communities effectively, indicating the need for improved dissemination strategies for awareness and treatment options.

Less decision-making power emerged as another critical barrier, directly impacting continued treatment-seeking. In Bangladesh, households are predominantly men-led, with men making most decisions [33, 34]. Shahabuddin et al. found that husbands’ decisions on professional maternal health care were influenced by their perceptions, beliefs, and knowledge of maternal health and resources [35]. A lack of accurate information among males delayed or incomplete treatment-seeking behavior in women. Strengthening awareness initiatives targeting men, alongside enhancing community-level outreach strategies, could address these barriers simultaneously, creating a supportive environment for women to complete fistula treatment.

### Systemic enhancement for treatment and strong regulations

#### Improve affordability of treatment cost

Unaffordability of treatment cost was significant challenges among participants to complete their treatment. Informal broker systems worsen financial strain by directing patients to costly private providers and unnecessary diagnostic tests. Many studies mentioned that financial barriers were significantly impact women’s treatment-seeking behavior [26, 36]. Adams et al. noted that brokers significantly increase patient expenses by diverting them away from free government care [25].

On the other hand, Bari et al. reported that subsidization for obstetric care was not associated with an increase in its utilization, suggesting that affordability does not necessarily improve access to care [37]. However, several participants delayed their treatment until they could save enough money to seek care. Most participants took out loans for hospitalization and diagnostic tests, which caused significant financial hardship.

In Bangladesh, out-of-pocket payments are the primary source of health funding, accounting for approximately 68.5% of total health expenditure in 2020, one of the highest rates in South Asia [38, 39]. The lack of dedicated funding for fistula treatment exacerbates this burden, as the government heavily relies on support from UNFPA and private donors [40]. The Bangladesh government must establish robust financial protection mechanisms for neglected diseases like fistula, ensuring equitable access to care. Implementing strict regulations against broker practices is also critical to alleviate these financial burdens.

### Establish national guidelines for treatment

Diagnostic delays and extended catheterization without national guidelines led to postponed treatment and mistrust in healthcare providers. A study in Kenya found similar issues, with missed diagnoses causing women to seek treatment at multiple hospitals [29]. Even though several women quickly sought treatment at hospitals, some doctors did not initially suspect fistula. Although various fistula diagnosis and treatment capacity-building initiatives have been implemented for healthcare providers in Bangladesh [41, 42], diagnostic failures have persisted, including in private hospitals. Capacity development programs, including private hospitals, should be strengthened as many cases initially involved them in surgeries and deliveries. However, this study focused on patient accounts, suggesting that some healthcare providers might have known the diagnosis, but did not communicate it clearly.

The treatment process, especially regarding medication and catheterization duration, lacked standardization in Bangladesh. Women experienced prolonged issues such as catheter leakage and painful burning sensations, which often led them to seek treatment at multiple hospitals, bypassing doctors’ advice, and ultimately eroding their trust in healthcare providers. Kees Waaldijk has demonstrated that continuous bladder drainage using a urethral catheter can successfully heal some fistula without surgery, potentially resolving 25% of suitable cases [43]. In Nigeria, national guidelines for urethral catheterization as a conservative treatment for fistula were developed in 2016, incorporating Waaldijk’s findings and detailing catheterization duration step by step [44]. Additionally, while traditional guidelines recommended waiting 3–6 months post-injury before performing surgery, there is growing support for early repair to reduce stigma and emotional distress for the patient [1, 45]. Although the optimal timing for surgery remains debated, prompt referrals to surgery-capable hospitals are essential to assess and determine the appropriate surgical timing. Establishing national treatment guidelines, especially preoperative preparation, is essential to ensure effective and proper fistula treatment.

#### Enhance effective referral and monitoring system

An ineffective referral system and lack of monitoring mechanisms to re-engage women who suspended treatment were revealed in the treatment-seeking path of women.

Participants did not report consulting community health workers when recognizing symptoms and seeking care. Despite the government’s introduction of “community-level identification mechanisms” to detect potential fistula cases [41], most participants relied on personal inquiries about surgical facilities or recommendations based on doctors' reputations. El Arifeen et al. highlighted Bangladesh’s success in healthcare through extensive community-based approaches, especially utilizing community health workers [46]. Leveraging these workers to integrate fistula detection with regular maternal and child health activities is essential. Further research should evaluate the effectiveness of these community-level systems.

Doctor referrals were often based on personal connections and typically involved only verbal recommendations or the written names of hospitals. Adams et al. noted that the absence of a structured referral system in Dhaka led to delays and poor health outcomes [25]. Structured referral systems with detailed treatment processes and diagnostic test information shared between the referred hospitals are necessary.

A monitoring system for tracking women with fistula would help to reintegrate them into treatment, especially when they are lost to follow-up. The United Nations emphasized the importance of systematic registration and tracking at community and facility levels [3]. Current systems lack the capacity to monitor the entire treatment process. Enhancing coordination between healthcare providers and community health workers would facilitate better tracking of women with fistula and more timely consultations, ultimately improving treatment adherence and outcomes.

### Medical communication with peers and emotional support

Women with fistula were unable to obtain sufficient information and counseling from their healthcare providers. Even when doctors diagnosed their condition, women often felt anxious due to a lack of information about their diagnosis and treatment process. Miscommunication between healthcare providers and patients negatively impacts patients’ understanding, treatment expectations, and level of hopefulness [47]. Effective communication and interpersonal skills from healthcare providers improve patient adherence, illness comprehension, and treatment outcomes [48]. Diagnosing and providing information about fistula is a sensitive process for both women and their families. In particular, obstetric fistula can cause chronic injuries such as renal failure, cervical destruction, rectal stenosis, and neurological injury [1]. Moreover, fistula is not always cured by a single surgery [45], even though the first surgery has the highest possibility of closing the abnormal hole [49]. Therefore, comprehensive medical communication and careful counseling are crucial for women with fistula and their families.

Peer support among women who have undergone fistula treatment is effective in encouraging treatment completion. Vowles et al. found that personal stories enhance message credibility, demonstrate the curable nature of fistula, foster hope, and motivate peers to seek treatment [27]. The United Nations has also emphasized the effectiveness of training fistula survivors as ambassadors to identify and refer women for treatment [3]. By listening to their peers' experiences, women with fistula can better understand the process and cope with challenges during the treatment-seeking process. Introducing opportunities for peer learning would facilitate treatment completion.

Emotional support from those around women is vital for maintaining their motivation to complete treatment. Women often face mental distress owing to persistent symptoms, treatment refusals, and negative community perceptions. However, support from close individuals helps sustain their motivation to complete treatment. Rajajan et al. emphasized that emotional and psychological support from treating physicians and families is crucial while waiting for surgical repair [50]. Healthcare providers should offer mental support and educate families on providing psychological support.

Ensuring that women remain motivated to seek treatment is crucial for treatment completion. Adequate medical communication, peer learning, and emotional support are essential for encouraging treatment-seeking behavior in women with fistula.

## Limitations

This study employed a facility-based approach, which inherently included only women with fistula who could access healthcare facilities for treatment. Women unable to reach these facilities were not captured in the study. Additionally, several potential biases may have affected the results. Recall bias impacted data accuracy due to the prospective nature of the study over an extended period. To mitigate this, data triangulation was adopted through family interviews, checking hospital records, and conducting interviews with participants at different times.

We also faced challenges in contacting participants multiple times because of issues such as their physical condition, absence from examinations, and reluctance to talk. The number and diversity of family IDIs were also limited due to the data collection period, including Ramadan and Eid, during which most families were absent from the NFC. Due to the limited data collection period, we faced challenges in determining the saturation point. However, this study deemed the saturation point to have been reached, as similar patterns in treatment-seeking paths were observed and primarily categorized into three groups.

## Conclusions

This study aimed to identify barriers and facilitators to completing fistula treatment and describe the patterns of treatment-seeking paths among women with genital fistula. Analysis of treatment-seeking paths revealed that women were missing the standardized treatment route, resulting in delayed treatment, hospital hopping, and treatment suspension due to various barriers. Women’s treatment-seeking processes were affected by environmental barriers rather than their own behavior, such as scarce information on illness and treatment in the community, less decision-making power, failure of medical communication, as well as systemic failures in the cost, treatment, and referral systems. Emotional, physical, financial, and peer support acted as facilitators motivating women to complete treatment. To ensure that women with fistula receive treatment through the appropriate route, raising awareness about fistula in society, enhancing the treatment and referral system, and providing medical communication along with peer and emotional support are highly recommended.

## Supplementary Information

Additional file 1.

## Data Availability

The transcripts and audio records of interviews analyzed during the current study are not publicly available due to concerns regarding participants' privacy and confidentiality.

## Abbreviations

DMCH: Dhaka Medical College Hospital
IDIs: In-depth interviews
KIIs: Key informant interviews
MOHFW: Ministry of Health and Family Welfare
NFC: National Fistula Centre
OGSB: Obstetrical and Gynecological Society of Bangladesh
PASS: Partners for Applied Social Sciences
PI: Principal investigator
UI: Uncertainly interval
UNFPA: United Nations Population Funds
WHO: World Health Organization

## Footnotes

1 “Practitioners of traditional medicine” such as Ayurvedic which is ancient medical practices that originated on the Indian subcontinent [51].

2 A therapy system developed by Samuel Hahnemann, based on treating diseases with minute doses of substances that cause similar symptoms in healthy individuals [51].

3 Water supplement upon which a verse from the Quran has been recited and blown.

4 Oil supplement upon which a verse from the Quran has been recited and blown.

5 The religious healer who uses verses from the Quran and spiritual practices for treatment [51]. In some places, people call *Hujur* a *Kabiraj*.

6 *Dalals* receive commissions for patient referrals. The practice of "commission-based referrals" is prevalent among private sector doctors, diagnostic center, and clinics, serving as a means to increase revenue through collaborative arrangements [25].
