# Supplementary figures and images for: Barriers and facilitators for treatment-seeking among women with genital fistula: a facility-based qualitative study in Bangladesh
Source: Trop Med Health. 2025 Feb 28;53:34. doi: 10.1186/s41182-025-00704-w (PMC11869737; doi:10.1186/s41182-025-00704-w)

# Additional File 1 Participants' treatment-seeking paths

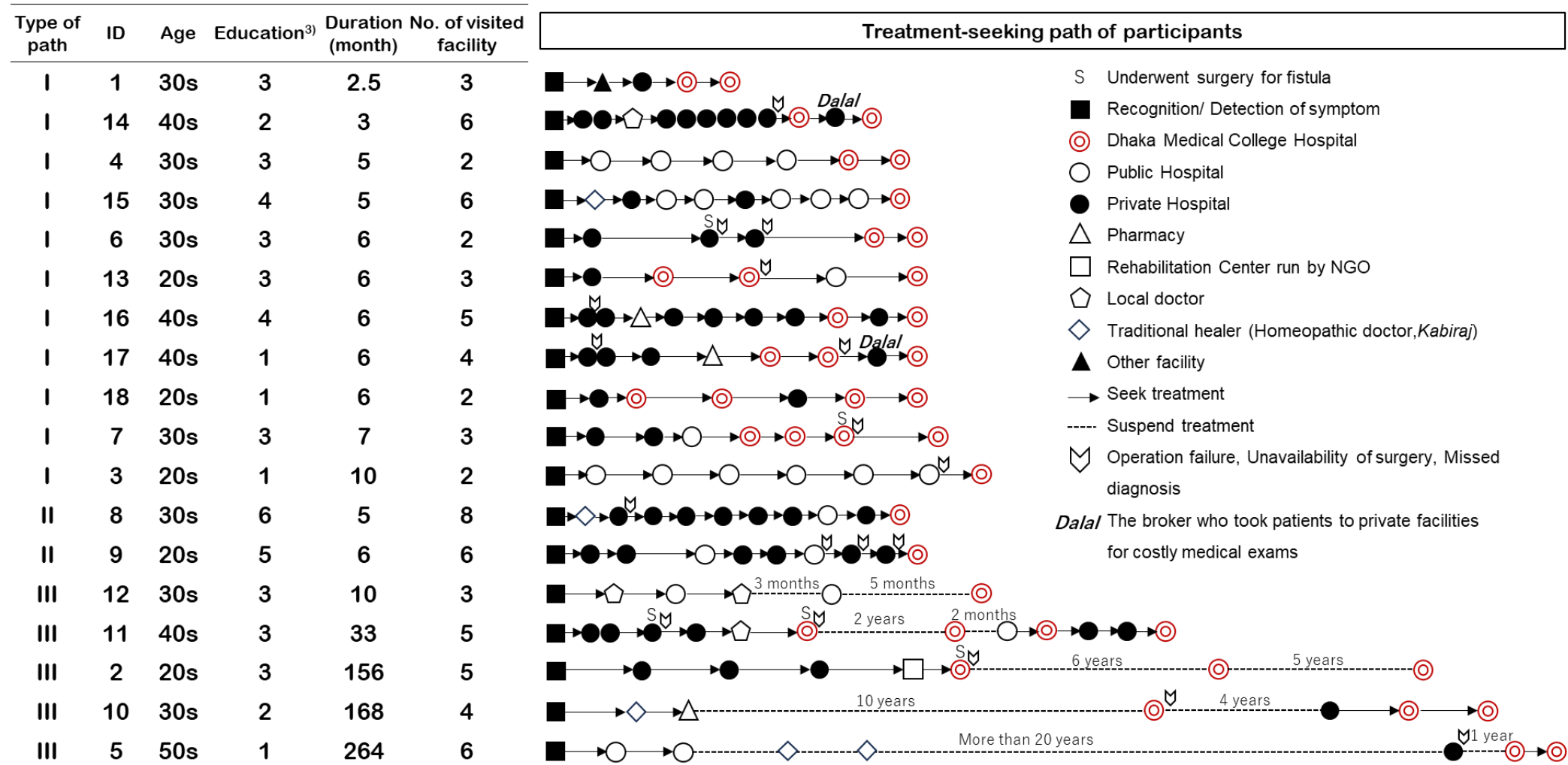

Supplement: Supplementary file 1 — Additional file 1. [file 41182_2025_704_MOESM1_ESM.pdf]
